# Impact of singular versus combinatorial environmental stress on RONS generation in *Drosophila melanogaster* larvae

**DOI:** 10.3389/fphys.2024.1426169

**Published:** 2024-08-29

**Authors:** Pratibha Bomble, Bimalendu B. Nath

**Affiliations:** ^1^ Stress Biology Research Laboratory, Department of Zoology, Savitribai Phule Pune University, Pune, India; ^2^ Department of Zoology, Indira College of Arts Commerce and Science, Pune, India; ^3^ MIE-SPPU Institute of Higher Education, Doha, Qatar

**Keywords:** oxidative stress, abiotic stressors, antioxidants, reactive oxygen and nitrogen species, *Drosophila*, aconitase

## Abstract

We investigated environmentally correlated abiotic stressor desiccation (D), heat (H), and starvation (S) in the generation of reactive oxygen and nitrogen species (RONS) using *Drosophila melanogaster* larvae as an experimental model, subjected to either individual stressors or exposed to a combinatorial form of stressors (D + H, H + S, and D + S). The study was also extended to find synergistic endpoints where the impacts of all three stressors (D + H + S) were exerted simultaneously. We estimated the lethal time (LT_20_) at specific doses using regression and probit analyses based on the larval survival. LT_20_ values were used as the base-level parameter for further oxidative stress experimental analysis work. First, all stressors led to the activation of a typical common oxidative stress-mediated response irrespective of the mode of exposure. As envisaged, *D. melanogaster* larvae exhibited a homeostatic stress tolerance mechanism, triggering an antioxidant defense mechanism, indicated by an elevated level of total antioxidant capacity and enhanced activities of superoxide dismutase, catalase, glutathione reductase, and glutathione peroxidase. In all types of stress-exposed regimes, we found a negative impact of stressors on the activity of mitochondrial enzyme aconitase. Elevated levels of other oxidative stress markers, viz., lipid peroxidation, protein carbonyl content, and advanced oxidative protein products, were obvious although the increment was treatment-specific. Desiccation stress proved to be the most dominant stressor compared to heat and starvation. Among the combination of stressors, rather than a single stressor, D + H impacted more than other binary stress exposures. Focusing on the impact of singular versus combinatorial stress exposure on RONS generation, we observed an increase in the RONS level in both singular and combinatorial forms of stress exposure although the magnitude of the increment varied with the nature of stressors and their combinations. The present study indicated an “additive” effect when all three stressors (D + H + S) operate simultaneously, rather than a “synergistic” effect.

## 1 Introduction

For the last couple of decades, climatic scenarios have intensified various environmental stressors. Fluctuations in environmental conditions pose a risk to the survival of living organisms, impacting their physiology of adaptation. In natural environments, living organisms periodically experience various abiotic stressors, such as heat, starvation, desiccation, hypoxia, and osmotic imbalance (Bijlsma and Loeschke, 2005; Bradley, 2009; Thorat et al., 2012; Thorat and Nath, 2018; Sokolova, 2021; Thorat et al., 2023). Insects, often considered barometers for environmental monitoring, have drawn the attention of stress biologists. Insects are routinely exposed to multiple environmental variations, thereby successfully occupying diverse ecological niches (Ramniwas et al., 2023; Thorat et al., 2023). Quite a few insect groups demonstrate remarkable tolerance, often selectively, toward abiotic stressors occurring either individually or simultaneously. One needs to choose either a stress-sensitive or stress-tolerant bioindicator insect model to investigate singular and multiple stress response mechanisms at the cellular, behavioral, and physiological levels (Hoffmann and Hercus, 2000; Holt and Miller, 2010).

A common stress-responsive biochemical signature observed in most abiotic stress conditions is the generation of reactive oxygen species (ROS), linking oxidative stress with desiccation, heat, and starvation (Malik and Storey, 2011; Thorat et al., 2016a; Zhang et al., 2019; Farahani et al., 2020; Miao et al., 2020; Pandey et al., 2020; Pati et al., 2024; Hermes-Lima et al 2015). Similar to ROS, reactive nitrogen species (RNS) are an additional set of free radicals and non-radical molecules generated under stress conditions, leading to nitrosative stress, and their significance is being revealed, mainly in plants (Del Río, 2015; Khan et al., 2023) and in pathophysiological circumstances in humans (Dalle-Donna et al., 2005; Wang et al., 2021). The generation of reactive oxygen and nitrogen species (RONS) occurs in many cell signaling pathways. Although many reports on RONS are available to date on plants exposed to multiple stressors, there are practically no studies carried out in non-human biota, especially in stress-bioindicator insects. Studies on RONS are warranted because the nitrosative stress responses can be exploited as a potential tool for biomonitoring environmental health.

The RONS generation follows species-specific strategies to combat singular and simultaneously acting multiple stressors. Recently, findings from our laboratory demonstrated a homeostatic response through RONS generation in a stress-resilient chironomid midge species, *Chironomus ramosus*, exposed to stressful fluctuations in temperature, humidity, and nutrient imbalances (Bomble and Nath, 2022). Having explored the effect of combinatorial stress on RONS generation in chironomid midge, an aquatic dipteran insect, we shifted our attention to drosophilid flies, which are an evolutionary distant and ecologically diverse dipteran terrestrial insect group. Among insects, the family Drosophilidae is one of the most well-investigated groups in stress biology, especially the popular model *Drosophila melanogaster*. The rationale for choosing *D. melanogaster* for the present study is its amenability to experimental manipulation. Previous work from our laboratory proved *D. melanogaster* not only as a stress-bioindicator species (Thorat et al., 2012) but also for laboratory-based simulation studies of environmental biotic and abiotic stress (Thorat and Nath, 2010; Thorat et al., 2016a; Thorat et al., 2016b). In the present paper, we investigated three abiotic stressors, desiccation (D), heat (H), and starvation (S), subjecting *D. melanogaster* larvae to each stressor individually or in combination, viz., either D + H, H + S, D + S, or D + H + S. We intended to know the status and level of attainment of antioxidant defense mechanisms in singular and combinatorial forms of stress exposure, and RONS served as the endpoints of our queries. Additionally, we explored the status of mitochondrial enzyme aconitase, which is a marker of stress at the cellular level (Gardner, 2002). This is the first report of RONS generation after combinatorial abiotic stress exposure in *D. melanogaster*, and the findings provided physiologically relevant insights.

## 2 Materials and methods

### 2.1 Rearing and maintenance of *Drosophila*

In this study, an inbred population of *D. melanogaster* (ORK strain) was maintained under controlled environmental conditions in a BOD incubator at 24°C ± 2°C. The *Drosophila* cultures were maintained in a nutrient-rich cornmeal–agar medium. To ensure the consistency of the experimental cohort, healthy early third-instar larvae were used for all experiments.

### 2.2 Stress treatment

#### 2.2.1 Experimental design

A total of 10 larvae of *D. melanogaster* were used in each experiment, in which the larvae were exposed to either a desiccation, heat, and starvation stressor or combinations of these stressors. LT_20_ (lethal time required for 20% mortality in the population) values were taken as an endpoint for each experiment (Supplementary Table S1).

#### 2.2.2 Desiccation

A measure of 500 g of silica gel was added to the desiccating chamber 12 h prior to its use in order to obtain <5% relative humidity (RH), which was monitored using a hygrometer. The larvae of *D. melanogaster* were desiccated in this chamber on dry tissue paper placed in a glass Petri dish. The LT_20_ values were taken as an endpoint for further experimental work. Untreated larvae were used as controls.

#### 2.2.3 Heat

Heat stress was administered by transferring the larvae of *D. melanogaster* to incubators set at 37°C. The induction of HSP70 occurs as a response to heat stress in *Drosophila* at 37°C (Lindquist, 1980) and hence 37°C was used for the experiment.

#### 2.2.4 Starvation

The larvae of *D. melanogaster* were removed from the rearing media, carefully cleaned, and then placed on wet tissue paper in a glass Petri plate without any nutrient medium. The starvation stress was applied at the LT_20_ time points (Supplementary Table S1).

#### 2.2.5 Multiple stressors

For combined stress treatments, the larvae were concurrently exposed to combinations of two or three stressors, including desiccation with heat (D + H), heat with starvation (H + S), and starvation with desiccation (D + S) or three stressors (D + H + S) simultaneously. The endpoint for each experiment was determined as the lethal time required for 20% mortality in the population (LT_20_). LT_20_ values were used as the base-level parameter across all treatments, enabling a comparison between singular and combinational stress effects. Each experiment was replicated thrice per treatment and conducted under controlled laboratory conditions.

### 2.3 Assay of oxidative stress markers

#### 2.3.1 Lipid peroxidation

The thiobarbituric acid reactive substance (TBARS) assay was conducted following the protocol outlined by Ohkawa et al. (1979), utilizing malondialdehyde (MDA) as the marker. A standard graph was plotted using 2 mM MDA, and calibration for subsequent dilutions was prepared accordingly to quantify MDA concentrations in the samples. For sample preparation, whole larvae of *D. melanogaster* (n = 80) from both treatment and control groups were homogenized in 1× phosphate-buffered saline (PBS) at specific time points. The resulting homogenates were centrifuged at 15,000 rpm for 30 min at 4°C to obtain the supernatant for further analysis. The supernatants were then mixed with a TBA reagent and subjected to a 60-min incubation in a boiling water bath alongside positive and negative controls, after which they were immediately transferred to ice for 10 min and then centrifuged at 8,000 rpm for 5 min. After centrifugation, the tubes were allowed to incubate at room temperature, and the absorbance was measured at 535 nm using a spectrophotometer. The concentration of MDA in the sample test was calculated in terms of μmol/mg of protein, providing a quantitative measure of lipid peroxidation levels.

#### 2.3.2 Protein carbonyl content

The quantification of protein carbonyl content involved its reaction with 2.4 dinitrophenyl hydrazine (DNPH), producing a protein–hydrazone complex quantified spectrophotometrically at 360 nm, adhering to the protocol outlined in the Protein Carbonyl Colorimetric Assay Kit (Item No. 10005020, Cayman Chemical). The control and treated sets of larvae (n = 50) were washed with 1× PBS to remove adherent particles. Subsequently, the larvae were homogenized in an ice-cold buffer (50 mM phosphate and 1 mM EDTA, pH 6.7) and centrifuged at 10,000 g for 15 min at 4°C to yield supernatants for further analysis. Nucleic acids were removed using 10% streptomycin sulfate. The assay mixture, comprising the sample, DNPH, 2.5 M HCl, and 20% trichloroacetic acid (TCA), was centrifuged at 10,000 g for 10 min at 4°C. The pellet was re-suspended in 1 mL of a 1:1 ethanol/ethyl acetate mixture and centrifuged at 10,000 g for 10 min at 4°C, and the whole process was repeated twice. Subsequently, the pellet was re-suspended in 500 μL of guanidine hydrochloride and centrifuged at 10,000 g for 10 min at 4°C. The absorbance of the supernatant was then measured at 360 nm using a UV spectrophotometer, facilitating the quantification of protein carbonyl content.

#### 2.3.3 Advanced oxidative protein products

The method described by Witko-Sarsat et al. (1996) was used to assess the levels of advanced oxidative protein products (AOPPs). Homogenates of larvae (n = 50) from both treated and control samples were incubated with potassium iodide in a rocking shaker at room temperature. Following this incubation period, ethyl acetate was added, and the spectrophotometric absorbance was measured at 340 nm. The absorbance values were normalized using a chloramine standard T-plot.

### 2.4 Assay for ROS generation

#### 2.4.1 Measurement of superoxide radicals

Following stress treatment, *D. melanogaster* larvae (n = 50) were homogenized in 1× PBS and subsequently centrifuged at 5,000 rpm at 4°C for 10 min. The resulting supernatants were then incubated in a light-protected environment with a 2′,7′-dichlorodihydrofluorescein diacetate (DCF-DA; D399, Invitrogen) solution for 30 min to detect the presence of superoxide radicals (O_2_^·−^). Post-incubation, the supernatants were analyzed using fluorimetry at wavelengths specific to the excitation and emission properties of the DCF-DA dye. A standard solution of DCF-DA dye was used, and the data were quantified in arbitrary fluorescence units.

#### 2.4.2 Measurement of hydrogen peroxide

The Amplex Red Hydrogen Peroxide Assay Kit (A22188, Invitrogen) was utilized to ascertain the concentration of hydrogen peroxide in both the control and treated samples of *D. melanogaster*, following the manufacturer’s guidelines. Post-stress treatment at respective time points, the larvae (n = 50) were homogenized in an assay buffer solution and subsequently centrifuged at 10,000 g for 15 min at 4°C. The resultant supernatant was carefully collected and combined with the reaction solution comprising the dye and horseradish peroxidase (HRP). The absorbance was measured at 560 nm using a microplate reader, and readings were normalized with a standard plot of H_2_O_2_.

### 2.5 Assay for RNS generation

#### 2.5.1 Measurement of the total nitrate/nitrite concentration

The total nitrate/nitrite concentration was quantified using the Total Nitrate/Nitrite Colorimetric Assay Kit (780001, Cayman Chemical), following the manufacturer’s protocol. Larvae from both the control and treated groups were homogenized in 1× PBS at pH 7.4 and subsequently centrifuged at 10,000 g for 20 min at 4°C to obtain the supernatant for further analysis. Nitrate/nitrite concentrations were determined by correlating the absorbance values with a standard plot of nitrate and nitrite concentration.

#### 2.5.2 Measurement of nitric oxide radicals

The concentration of nitric oxide radicals was assessed using the fluorescent dye 4,5-diaminofluorescein diacetate (DAF-2DA; ab145283, Abcam), which is specific for reactive nitrogen species. Following stress treatment, the larvae were homogenized in 1× PBS and subsequently centrifuged at 5,000 rpm at 4°C for 10 min to obtain the supernatant. The supernatant was then incubated in the dark with a 4,5-DAF-2DA (Abcam) solution for 20 min to enable the detection of nitric oxide radicals. Post-incubation, the supernatants were analyzed by fluorimetry at dye-specific excitation and emission wavelengths (Ex/Em = 491/513). The DAF-DA dye solution was run as the standard, and the data were represented in arbitrary fluorescence units.

### 2.6 Estimation of the total RONS concentration

Total RONS generation was quantified using the ROS-ID^®^ Total ROS Detection Kit (ENZ-51011, Enzo Chemicals) in the larval hemolymph of *D. melanogaster* from both the control and treated groups, following the manufacturer’s protocol. The Total ROS Detection Kit includes the oxidative stress detection reagent (Green, Ex/Em 490/525 nm) to directly monitor the reactive oxygen and/or nitrogen species (ROS/RNS). This non-fluorescent, cell-permeable detection probe reacts directly with a wide range of reactive species (hydrogen peroxide, peroxynitrite, and hydroxyl radicals), yielding a green fluorescent product. The samples were analyzed by fluorimetry using a microplate reader (FLUOstar Optima, BMG LABTECH, Germany) at dye-specific excitation and emission wavelengths (Ex/Em = 490/525 nm). Hemocytes from *D. melanogaster* were isolated using the cuticle puncture method. In brief, the larvae were carefully removed from the control and treated groups and thoroughly washed with Ringer’s solution. The hemolymph was then collected from the larvae by bleeding into 1× PBS in a dissecting glass, and the collected hemolymph from both the control and treated groups was transferred into microcentrifuge tubes with Schneider’s insect medium, and this sample was used for further analysis. The data obtained were represented in the form of arbitrary fluorescence units, providing a quantitative measure of the total RONS generation.

### 2.7 Microscopic study to investigate the status of reactive species

#### 2.7.1 Salivary glands of *Drosophila melanogaster* larvae

Salivary glands from both control and treated early third-instar larvae of *D. melanogaster* were dissected and fixed in 4% paraformaldehyde after undergoing permeabilization treatment, preparing them for further study and analysis.

#### 2.7.2 Assay for the visualization of ROS and RNS generation

Intracellular ROS were analyzed by fluorescence microscopy using 2′,7′-DCF-DA (Cayman Chemical). The differential interference contrast (DIC) image was used to visualize the structure of the tissue, and 4′,6-diamidino-2-phenylindole (DAPI), a fluorescent stain that binds strongly to A-T-rich regions in DNA, is used to label the nuclei of the cell; here, we used DAPI for counterstaining. The dissected salivary glands of *D. melanogaster* from both the control and treated groups were incubated with 2′,7′-DCF-DA (Abcam) dye to detect ROS and 4,5-DAF-2DA (Abcam) dye to detect RNS for 30 min, followed by counterstaining with DAPI for 5 min in the dark. The samples were then examined under a fluorescence microscope (Carl Zeiss), utilizing appropriate filters for DCF-DA (Ex/Em = 492–495/517–527), DAF-DA (Ex/Em = 491/513), and DAPI (Ex/Em = 358/461).

### 2.8 Estimation of aconitase enzyme activity

Mitochondria were extracted from early third-instar larvae of *D. melanogaster*, sourced from both the control and treated groups, utilizing the procedure outlined by Wen et al. (2016), with some adaptations. Aconitase activity was quantified spectrophotometrically using Thermo Fisher Scientific instruments, where NADPH formation was tracked at 340 nm using the coupled assay method developed by Gardner (2002). The isolated mitochondria served as the subcellular fractionated samples for the assessment of aconitase enzyme activity. Spectrophotometric measurement of aconitase activity was conducted using Thermo Fisher Scientific instruments, with NADPH formation monitored at 340 nm. The samples were then introduced into an assay buffer (comprising 50 mM Tris–HCl at pH 7.4, 0.6 mM MnCl_2_, 5 mM sodium citrate, 0.2 mM NADP^+^, 0.1% v/v Triton X-100, and 0.4 units/mL isocitrate dehydrogenase (Sigma) pre-equilibrated to 30°C). Each sample was assayed in quadruplicate, and readings were taken at 15-s intervals over 7 min. The resulting linear slopes were averaged to derive the measurement of aconitase activity for each sample.

### 2.9 Measurement of antioxidant enzymes

#### 2.9.1 Preparation of the homogenate for antioxidant assay

To prepare the larval sample for antioxidant assay, larvae from the control and treated groups of *D. melanogaster* were homogenized in a protein extraction buffer, which contained 1 mM phenyl methyl sulfonyl fluoride (PMSF), 1 mM ethylenediaminetetraacetic acid (EDTA), 50 mM phosphate buffer (pH 7.2), and 0.1% Triton X-100. The buffer was maintained under chilled conditions until use. Larvae were added to the chilled buffer, homogenized, and then centrifuged at 14,000 rpm for 30 min at 4°C. This homogenate was used as a crude extract for determining the enzyme activity. The amount of protein obtained from the above homogenate was quantified using the Bradford method, with bovine serum albumin as the standard.

#### 2.9.2 Superoxide dismutase assay

By using the principle described by Beauchamp and Fridovich (1971), the specific activity of superoxide dismutase (SOD) (EC 1.15.1.1) was determined by measuring its ability to inhibit the photochemical reduction of nitro blue tetrazolium (NBT) chloride. The reaction mixture consisted of 100 mM KPO_4_ buffer, pH 7.8, 0.01 μM EDTA, 65 mML methionine, 750 μM NBT chloride, 2 mM riboflavin, and 50 μL of enzyme extract in a total volume of 3 mL. Riboflavin was added at the end, and the tubes were mixed by shaking. Two sets of the above reaction mixture were prepared: one set was kept under light conditions (20 W) while the other was kept under dark conditions for 30 min. Similarly, mixtures without the enzyme extract were kept under light and dark conditions and used as controls. Absorbance was measured at 560 nm. One unit of SOD activity (U) was defined as the amount of enzyme required to cause 50% inhibition of the photoreduction rate of NBT. The results were expressed as unit activity (U)/mg of protein.

#### 2.9.3 Catalase assay

The specific activity of the catalase (CAT) (EC 1.11.1.6) enzyme was measured by using the principle of its ability to convert H_2_O_2_ to a product, as described by Aebi (1983). The reaction mixture consisted of 100 mM phosphate buffer, pH 7.0, 20 mM H_2_O_2_, and 50 μL enzyme extract in a total volume of 1 mL in quartz cuvettes. The readings were taken at 240 nm at intervals of 30 s for 3 min to check changes in the amount of H_2_O_2_ using a spectrophotometer. One unit of enzyme was defined as the amount of enzyme required to convert 1 mol of H_2_O_2_ to a product in 1 s. The results were expressed in unit activity (U) per microgram (mg) of protein.

#### 2.9.4 Glutathione reductase assay

The specific activity of glutathione reductase (GR) (EC 1.8.1.7) was determined by monitoring its ability to oxidize NADPH, as described by Goldberg and Spooner (1983). The assay mixture consisted of 100 mM phosphate buffer, pH 7.2, 0.17 mM NADPH, 0.5 mM EDTA, 2.2 mM oxidized glutathione, and 100 μL of enzyme extract in a total volume of 1 mL. All components were mixed properly, and the rate of oxidation of NADPH was monitored up to 5 min at intervals of 30 s by measuring the absorbance at 340 nm. The enzyme activity was calculated in terms of U/mg of protein.

#### 2.9.5 Glutathione peroxidase assay

To determine the specific activity of glutathione peroxidase (GPx) (EC 1.11.1.9), we used the method proposed by Lawrence and Burk (1976). For this assay, the reaction mixture consisted of 100 mM KPO_4_ buffer, pH 7.0, 1 mM NaN_3_, 0.2 mM NADPH, 1 U GR, 1 mM GSH, 0.25 mM H_2_O_2_, 1 mM EDTA, and 100 μL of enzyme extract in a total volume of 1 mL. Absorbance was measured at 340 nm for 5 min at an interval of 30 s. Enzyme activity was measured in terms of U/mg of protein.

#### 2.9.6 Total antioxidant capacity

The antioxidants can inhibit the oxidation of 2,2′-azino-di-3-ethylbenzothiazoline sulfonic acid (ABTS) by metmyoglobin, and based on this, the absorbance was taken at 405 nm, according to the manufacturer’s protocol (709001, Cayman Chemical). The capacity of the antioxidants in the sample to prevent ABTS oxidation was compared with that of Trolox, a water-soluble tocopherol analog, and was quantified as molar Trolox equivalents. The control and experimental larvae of *D. melanogaster* were homogenized in 100 μL of the extraction buffer (provided with a kit) and then centrifuged at 12,000 rpm for 15 min at 4°C, while the supernatants were used for further experiments. A standard curve was prepared using different concentrations of Trolox for calculating the total antioxidant capacity of samples.

### 2.10 Statistics

Different parameters were monitored in normal and stress-exposed third-instar larvae of *D. melanogaster.* An analysis of variance was carried out to find the significant differences in the means, considering each endpoint as a dependent variable and the duration of exposure (LT_20_ of each stressor and combination of stressors) as independent variables. The data were represented as the mean ± SD. The *post hoc* test was used for the statistical comparison between the groups, and Tukey’s multiple comparisons test was carried out to determine statistical significance within the groups. A value of *p* < 0.05 was considered statistically significant (SPSS, version 12.0, SPSS Inc., Chicago, IL, United States).

## 3 Results

### 3.1 MDA, AOPP, and protein carbonyl content measurement

**FIGURE 1:**
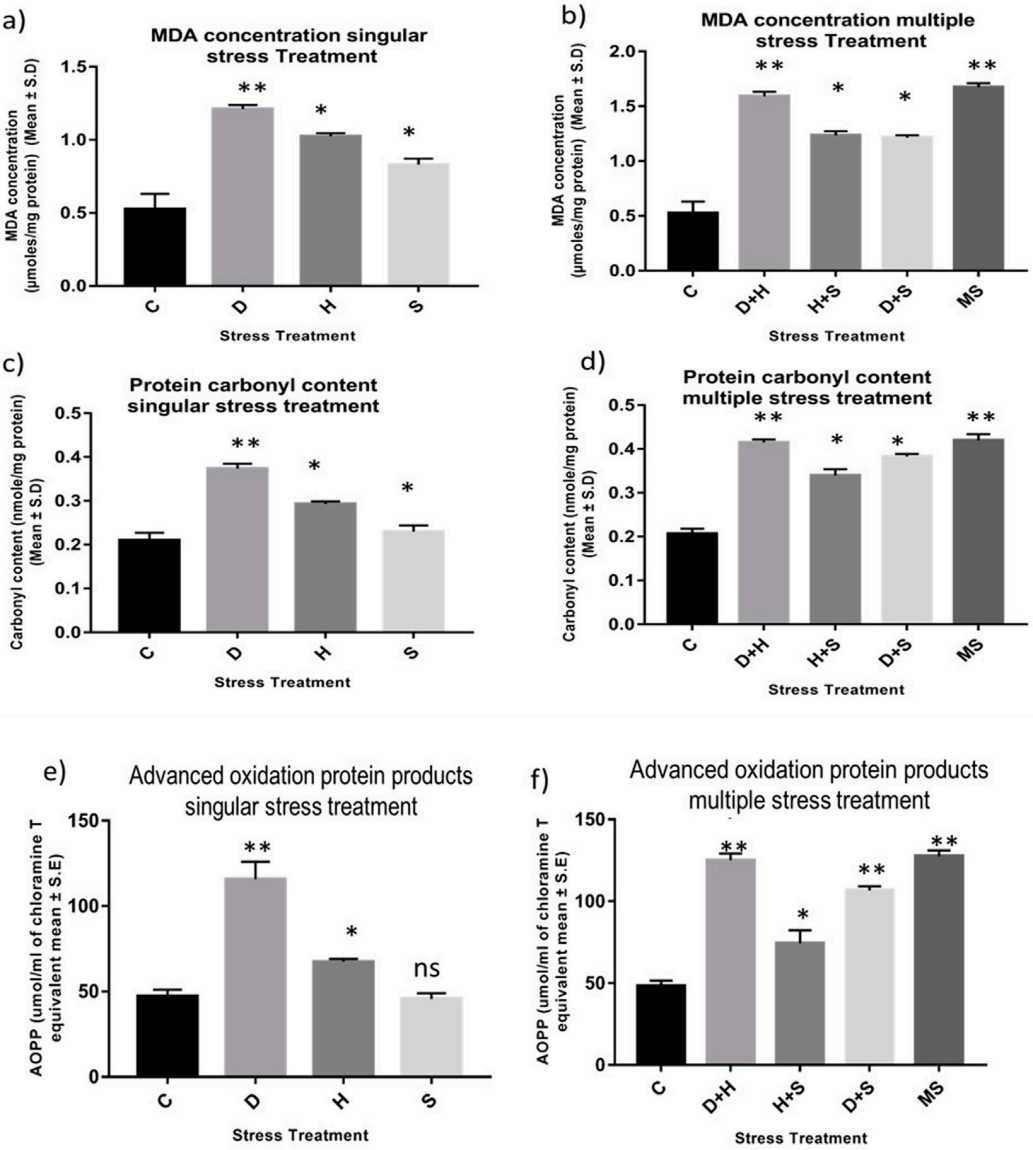
Impact of oxidative stress: Spectrophotometric measurement of **(A, B)** Lipid peroxidation (mean ± SD U/mg protein) **(C, D)** Protein Carbonyl content (mean ± SD U/mg protein) **(E,F)** AOPP (mean ± SD U/mL) in third instar larvae of *D. melanogaster*. Values represent mean and the vertical bars represent SD. Data shown are representative of three independent experiments. *** *P* <0.001; ** *P* < 0.01; * *P* < 0.05 indicates level of significance.

An increase in the concentration of MDA was evident in the treated larvae compared to the untreated larvae. The increase in the concentration of MDA was more significant in the case of heat stress with desiccation stress than in other combinatorial forms of stressors. When stressors are present in singular form, the increment in the level of MDA was more prominent in the case of desiccation than in the other two stressors, heat stress and starvation stress. The MDA concentration reached a maximum when all three stressors (MS) acted on the larvae simultaneously (Figures 1A, B). Protein carbonyl content is a known marker of protein oxidation, mainly caused by the highly reactive free radical species. The increase in the level of protein carbonyl content with stress treatment was conspicuous, but this increase was more prominent in the case of desiccation than in other singular stressors like heat stress and starvation stress (Figure 1C). The results from combinatorial studies revealed that desiccation stress in combination with heat stress was more vulnerable than desiccation alone and also in other forms of combination. The impact of D + H apparently was equivalent to MS (Figure 1D). AOPPs are toxins formed through the reaction of chlorinated oxidants with proteins during oxidative stress. Here, an increase in the concentration of AOPP in desiccation-stressed larvae was observed compared to heat and starvation-stressed counterparts. Starvation stress did not exert its effect on the AOPP level, and the larvae maintained a similar level as observed in the control (Figure 1E). This increase was more evident in the combinatorial form (H + D) than in the form of singular stressors. The AOPP level was high, as observed in the case of D + H, when all three stressors (MS) acted on the larvae simultaneously (Figure 1F).

### 3.2 Quantification of ROS: superoxide radicals and hydrogen peroxide

**FIGURE 2:**
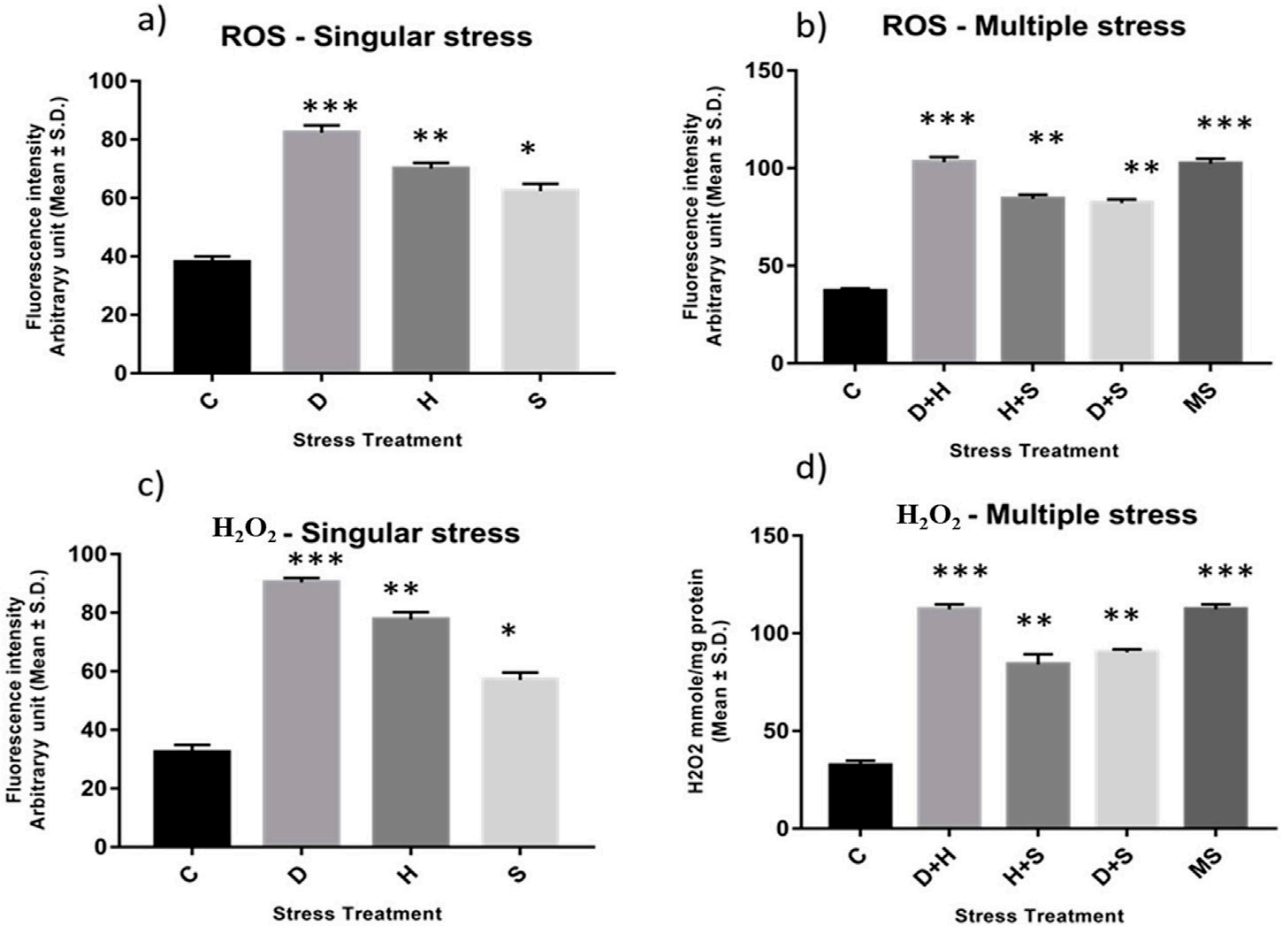
Spectrofluorometric measurement of ROS; **(A, B)** superoxide radical expressed as level of arbitrary unit (mean ± SD) **(C, D)** Quantification of Hydrogen peroxide nmole/mg of protein (mean ± SD) in third instar larvae of *D. melanogaster*. Values represent mean and the vertical bars represent SD. Data shown are representative of three independent experiments. *** *P* < 0.001; ** *P* < 0.01; * *P* < 0.05 indicates level of significance.

An increase in the production of ROS resulting from stress induced an imbalance between oxidants and antioxidants, as evident from the enhanced level of superoxide radicals and hydrogen peroxide, along with the stress exposure, compared to the control. In singular form, the increase was more prominent in the case of desiccation stress than that in heat stress and desiccation stress. This increase was more evident under combinatorial and multiple stress conditions desiccation combined with heat stress (D + H) was more potent than desiccation combined with starvation stress (D + S) and heat combined with starvation stress (H + S). In the presence of more than one stressor, D + H impacted the same way when all three stressors (MS) acted simultaneously (Figures 2A–D).

### 3.3 Reactive oxygen species imaging analysis

**FIGURE 3:**
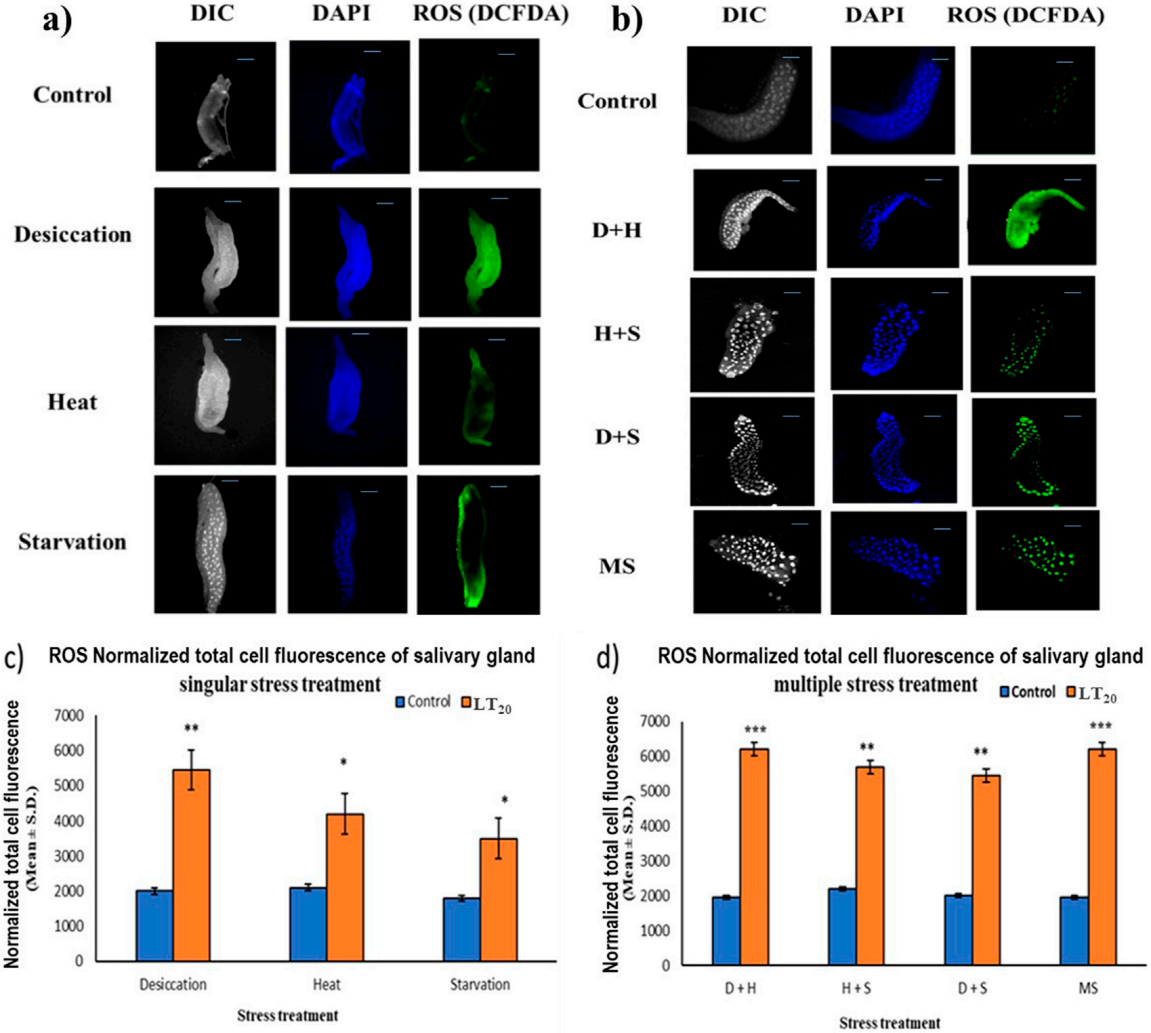
Microscopic visualization of ROS. Fluorescence microscopic images of *D. melanogaster* exposed to desiccation, heat, starvation, D + H, H + S, and D + S stress treatments compared against control salivary glands, following **(A)** singular stress and **(B)** combinatorial stress treatments to visualize reactive oxygen species. Normalized cell fluorescence intensity of **(C)** singular and **(D)** combinatorial stress treatments to quantify reactive nitrogen species. ***p < 0.001; **p < 0.01; and *p < 0.05; ns: non-significant. Scale bar: 200 µm. **(A,B)** Differential interference contrast panel for the ease of resolving the outline.

In fluorescence microscopic examination, the generation of ROS was validated in a metabolically active glandular tissue, the salivary glands of *D. melanogaster* larvae, and the ROS was quantified by green fluorescence intensity generated by the indicator DCF-DA dye. An increase in fluorescence intensity was observed with each stress treatment compared to the control salivary gland dissected from larvae without stress exposure. Salivary glands from desiccated larvae showed more green fluorescence intensity, indicative of higher levels of ROS in multiple stress treatments (Figures 3B, D) than in exposure to individual stressors (Figures 3A, C). When comparing the impact of singular stressors, desiccation stress generated more ROS than heat and starvation (Figures 3A, C). For a multiple stress regime, D + H and MS showed similar levels of elevation (Figures 3B, D).

### 3.4 Quantification of RNS: nitric oxide radical and nitrite/nitrate concentration

**FIGURE 4:**
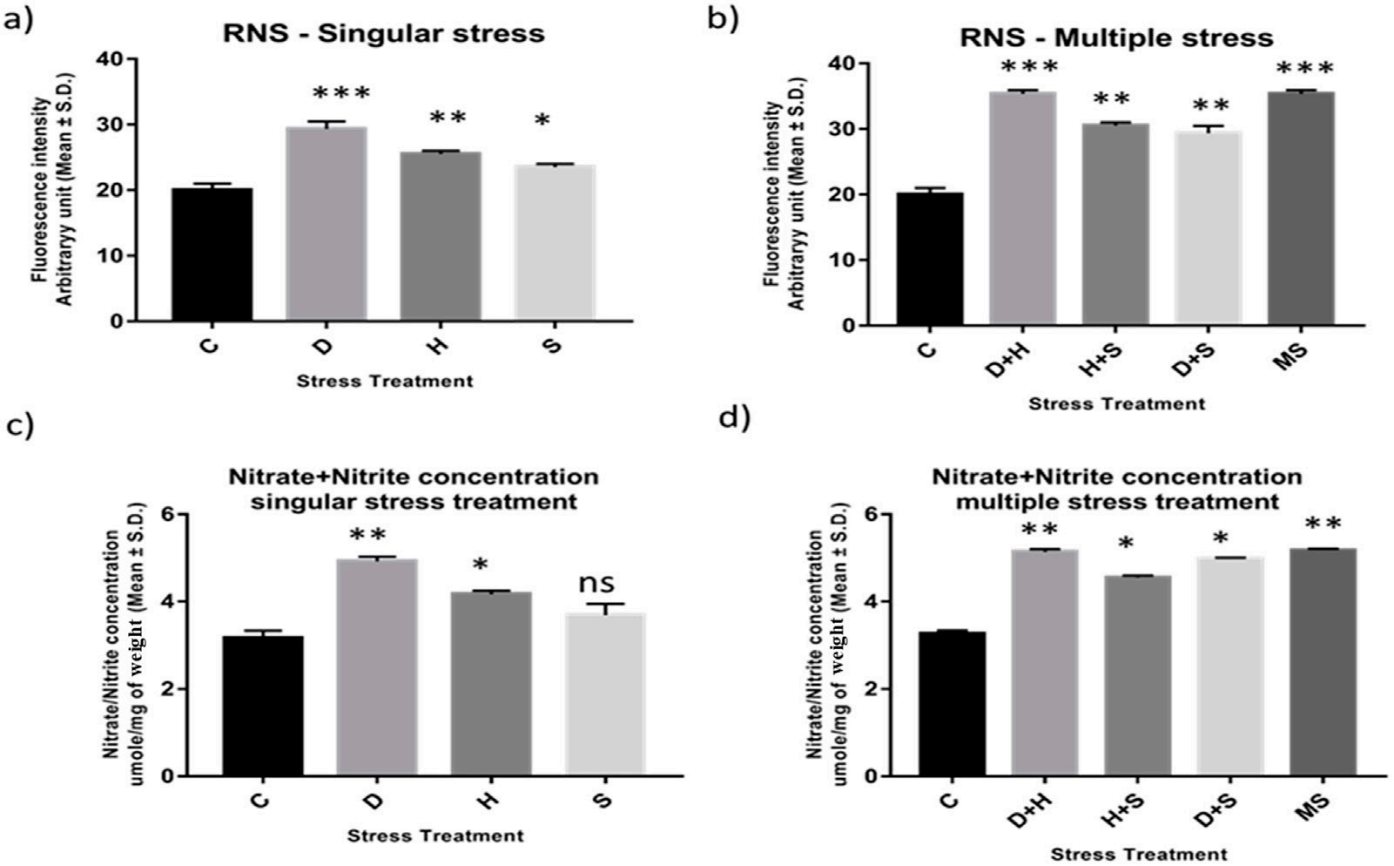
**(A, B)** Estimation of level of nitric oxide radical Arbitrary unit (mean ± SD) in third instar larvae of *D. melanogaster*. **(C, D)** Determination of Total Nitrate/Nitrite concentration umole/mg of weight (mean ± SD) in fourth instar larvae of *D. melanogaster*. Values represent mean and the vertical bars represent SD. Data shown are representative of three independent experiments. *** *P* < 0.001; ** *P* < 0.01; * *P* < 0.05 indicates significance level.

Abiotic stress administered in the present study induced oxidative stress, leading to the generation of nitrated species. RNS and the ratio of nitrate/nitrite concentration were all found to be elevated in the treated larvae of *D. melanogaster*. The treated larvae showed a higher ratio of nitrate/nitrite concentration and nitric oxide radicals than the corresponding control ones. While the increase was more prominent in the case of desiccation-stressed larvae than in the other singularly exposed larvae, the combinatorial exposure showed an increment notable in the case of heat with desiccation compared to other combinations. The consequences of D + H were similar to the impact of all three stressors (MS) on the larvae (Figures 4A–D).

### 3.5 Reactive nitrogen species imaging analysis

**FIGURE 5:**
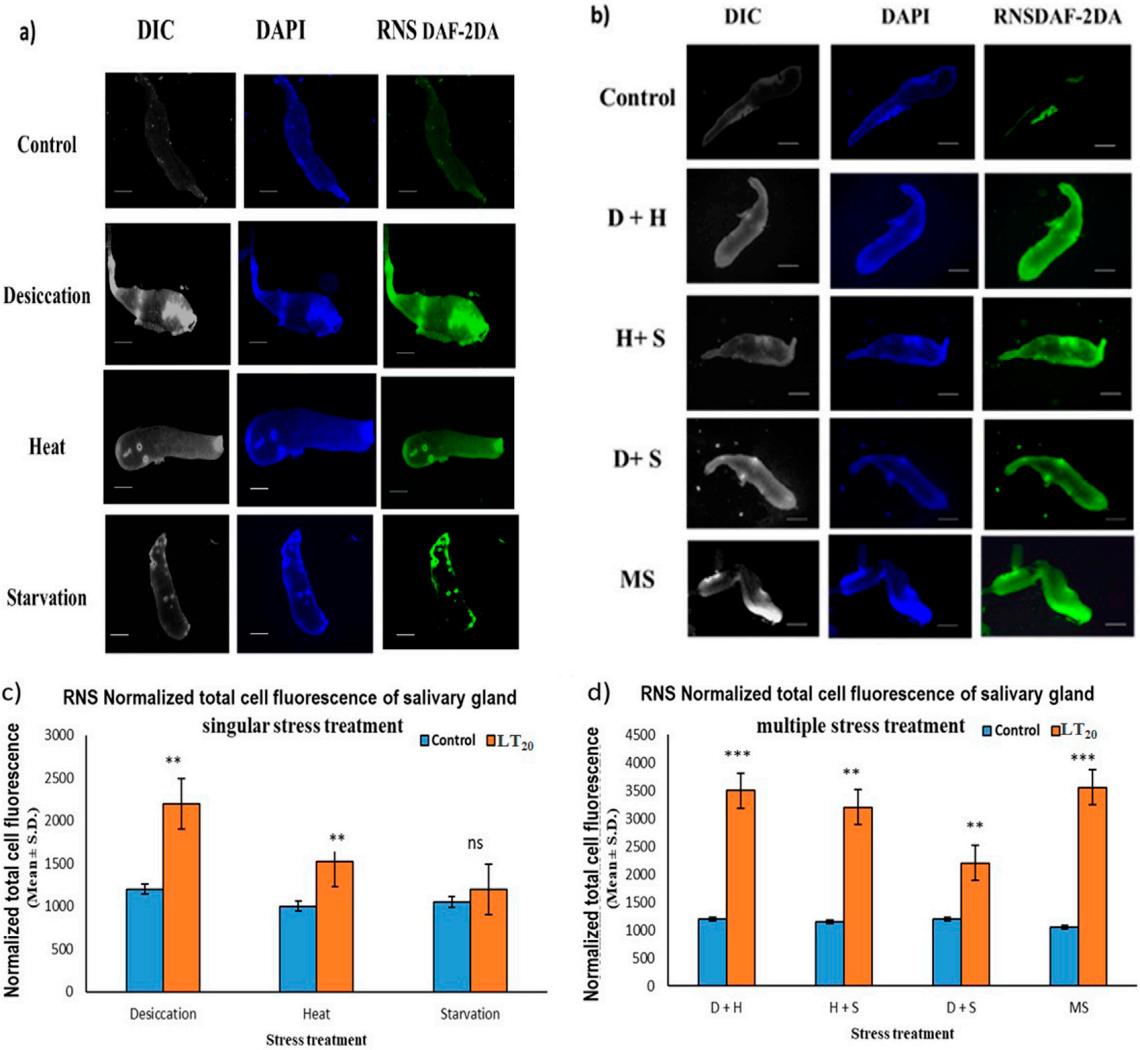
Fluorescence microscopic visualization of RNS: salivary gland images of *D. melanogaster* exposed to stress compared against the control with larvae subjected to **(A)** singular and **(B)** combinatorial stress treatments to visualize RNS. Normalized cell fluorescence intensity of **(C)** singular and **(D)** combinatorial treatment of salivary glands to quantify RNS. ***p < 0.001; **p < 0.01; and *p < 0.05 indicate significance levels. Scale bar: 200 µm. **(A,B)** DIC panel for the ease of resolving the outline.

The increase in the intensity of green fluorescence resulting from the marker DAF-2DA dye was an indication of the presence of nitric oxide radicals. The metabolically active glandular tissue of the salivary glands of *D. melanogaster* was exposed to stress treatments as carried out in previous experiments, and the results showed an increase in green fluorescence intensity in the stressed larvae compared to the control larvae. The higher green fluorescence intensity was an indication of the elevated level of reactive species in the desiccated salivary glands than in the glands of the starved and heat stressed larvae. In combinatorial form, glandular tissues of the larvae subjected to desiccation combined with heat stress treatment showed higher fluorescence (Figures 5A–D).

### 3.6 Measurement of the total RONS and aconitase enzyme activity

**FIGURE 6:**
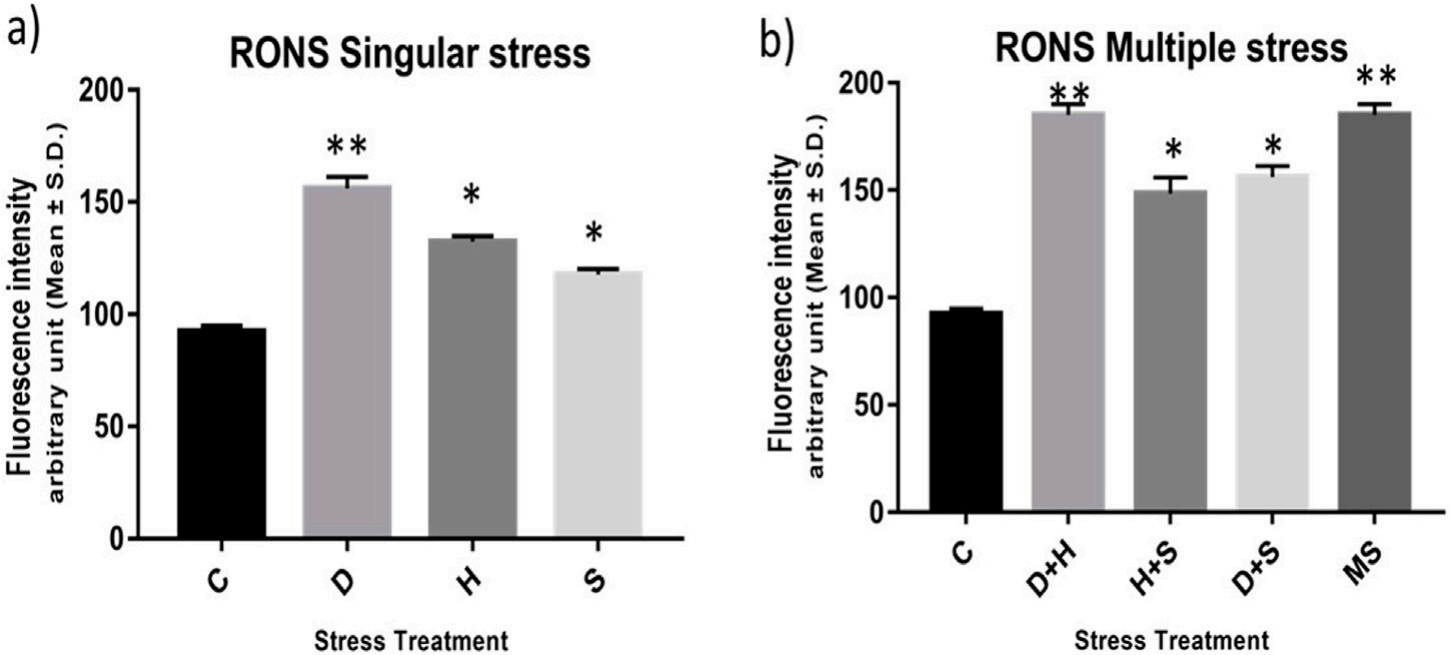
**(A,B)** Measurement of the total RONS fluorescence intensity arbitrary unit (mean ± SD) in third-instar larvae of *D. melanogaster*. Values represent the mean, and the vertical bars represent SD. Data shown are representative of three independent experiments. ***p < 0.001; **p < 0.01; and *p < 0.05 indicate significance levels.

**FIGURE 7:**
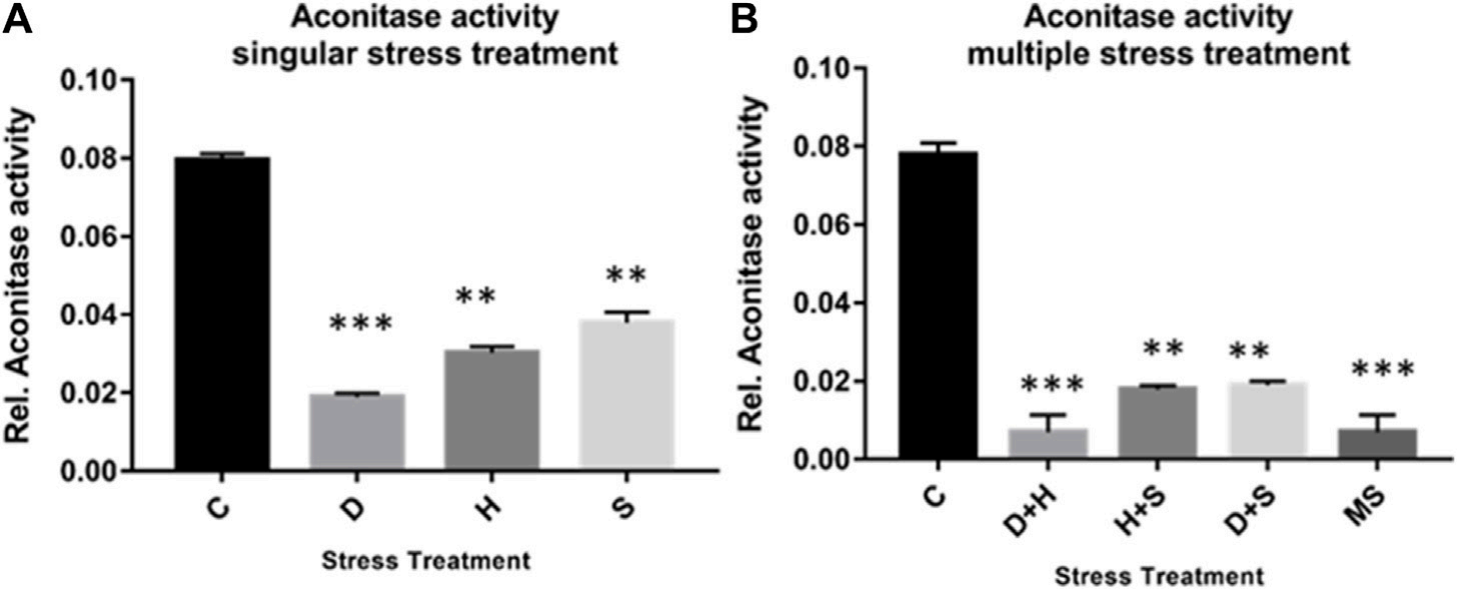
**(A,B)** Mitochondrial aconitase activity relative to the control with stress treatment (mean ± SD) in third-instar larvae of *D. melanogaster*. Values represent the mean, and the vertical bars represent SD. Data shown are representative of three independent experiments. ***p < 0.001; **p < 0.01; and *p < 0.05 indicate significance levels.

The result of the collective measurement of RONS like hydrogen peroxide, peroxynitrite, and hydroxyl radicals showed the generation of total RONS, and it was more evident in the case of desiccation stress and also in combination with heat stress (Figures 6A, B). The quantification of mitochondrial aconitase enzyme activity showed that the concentration of the superoxide radical was inversely proportional to aconitase enzyme activity. Interestingly, the decrease in relative aconitase enzyme activity was more conspicuous in desiccated larvae than in the other singular stressors, while in combinations of stressors, it was more evident in D + H stress than in other combinations. As observed in other sets of experiments, the D + H impact resembled that of MS, where all stressors act together (Figures 7A, B).

### 3.7 Measurement of antioxidant enzyme activity

**FIGURE 8:**
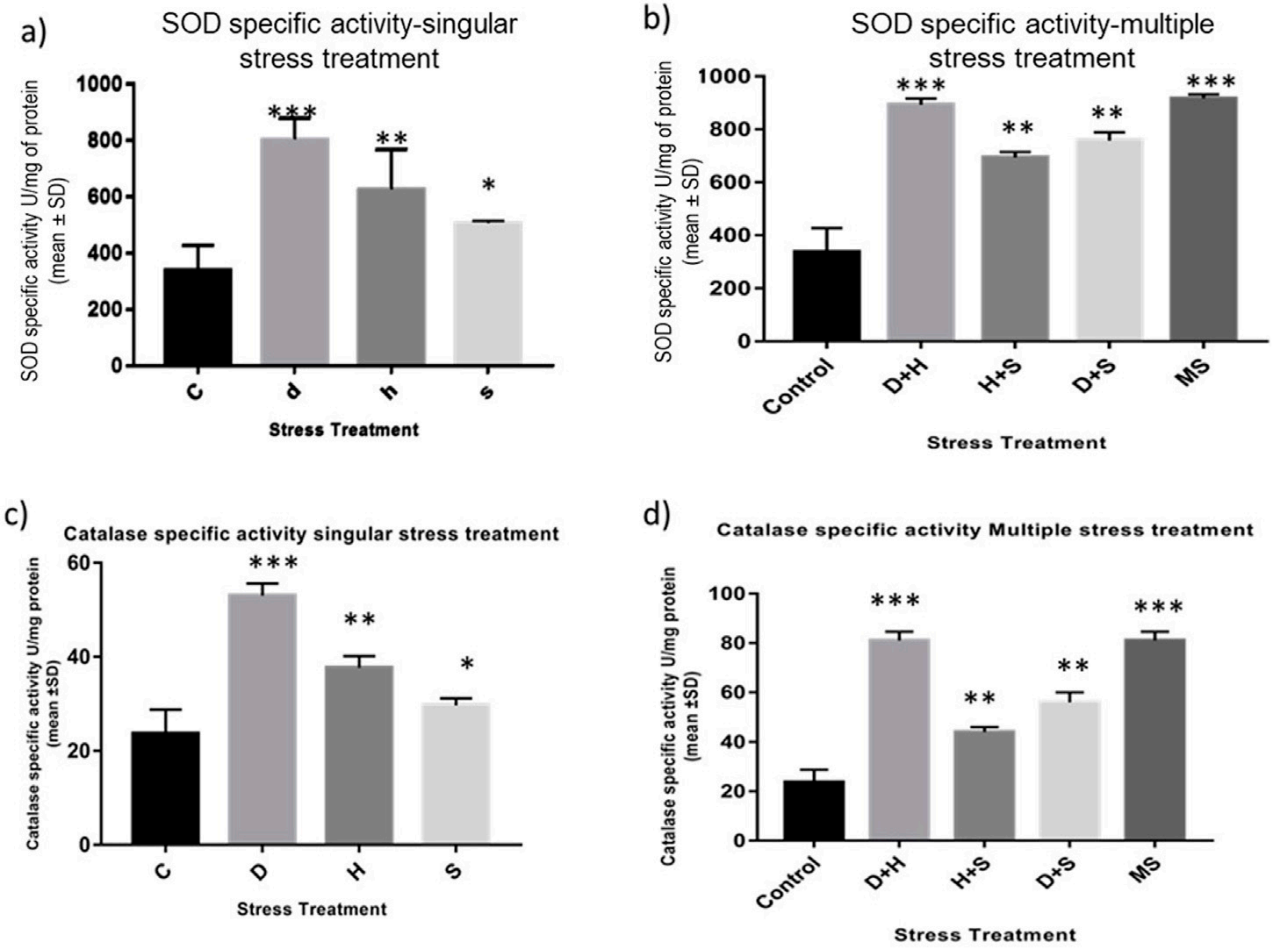
**(A,B)** Spectrophotometric measurement of **(A,B)** superoxide dismutase activity and **(C,D)** catalase activity of the whole larvae of *D. melanogaster* exposed to desiccation, heat, starvation, D + H, H + S, and D + S stress treatments compared with control groups and between treated groups. ***p < 0.001; **p < 0.01; and *p < 0.05 indicate significance levels.

**FIGURE 9:**
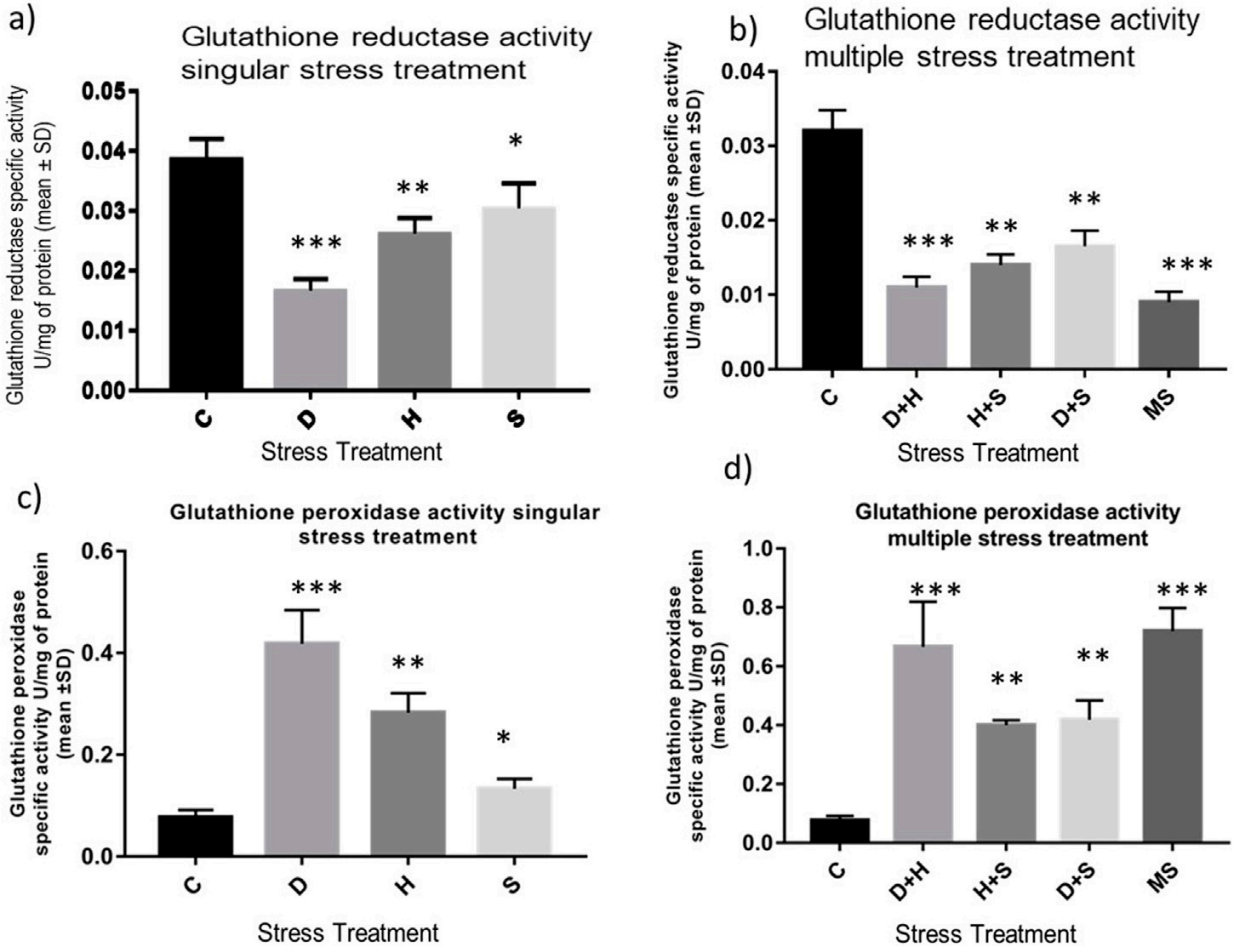
Determination of change in the activity of **(A,B)** glutathione reductase and **(C,D)** glutathione peroxidase of the whole larvae of *D. melanogaster* exposed to desiccation, heat, starvation, D + H, H + S, and D + S stress treatments compared with control groups and between treated groups. ***p < 0.001; **p < 0.01; and *p < 0.05 indicate significance levels.

**FIGURE 10:**
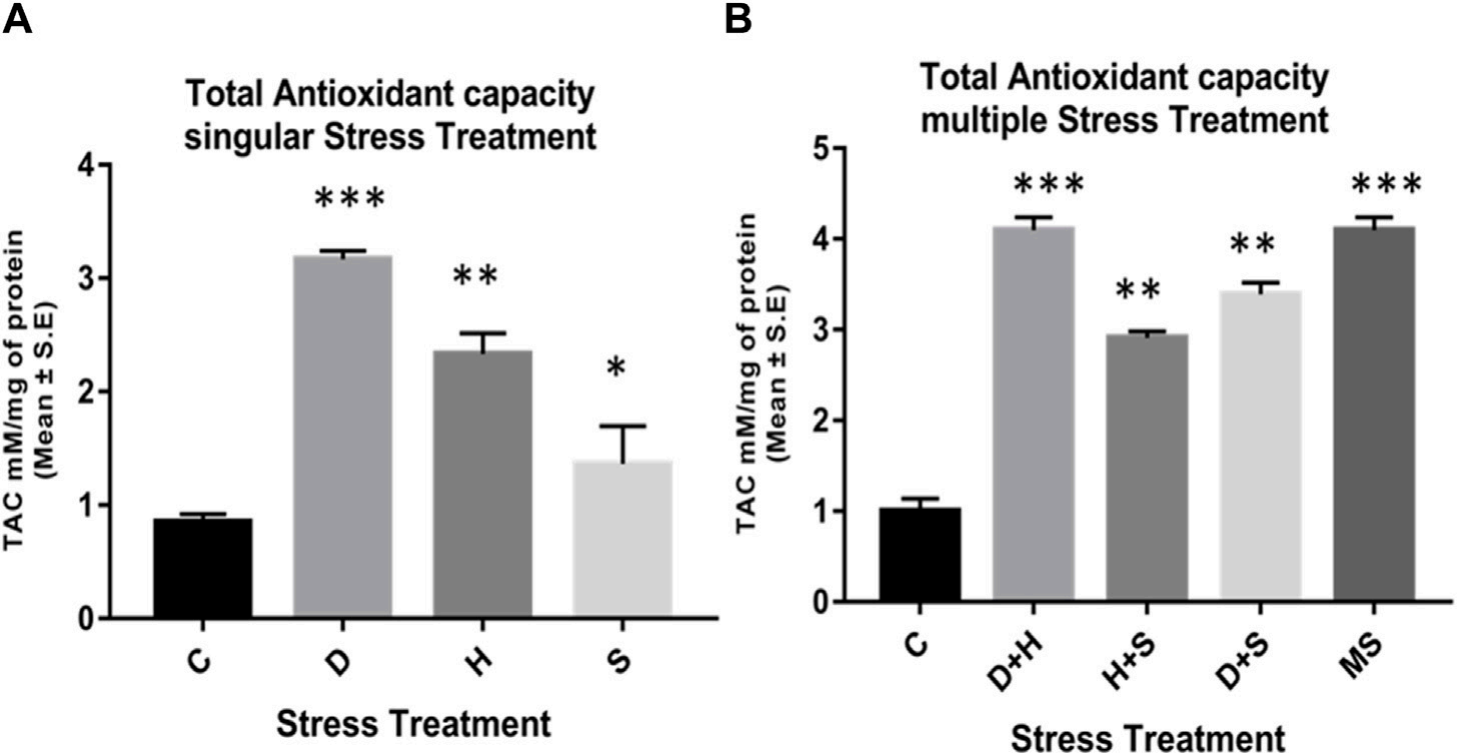
Quantification of the total antioxidant capacity of the whole larvae of *D. melanogaster* exposed to **(A)** singular and **(B)** multiple stress treatments compared with control groups and between treated groups. ***p < 0.001; **p < 0.01; and *p < 0.05 indicate significance levels.

The data obtained from the quantification of antioxidant enzyme activity revealed that the specific activity of superoxide dismutase, catalase (Figures 8A–D), and glutathione peroxidase (Figures 9C, D) was directly proportional to stress treatment, while the specific activity of glutathione reductase was inversely proportional to stress treatment (Figures 9A, B). It was evident that the increase in the specific activity of SOD and catalase was more in the case of desiccation stress than that of heat stress and starvation stress (Figure 8A), while in combinatorial form, it was quite evident that the incremental change was prominent in the case of D + H compared to the other two combinations D + S and H + S (Figure 8B). Catalase showed a similar increase in activity with respect to stress treatments either in singular or multiple stress regimes (Figures 8C, D). Nevertheless, the total antioxidant capacity showed an increase in respective stressor-exposed larvae compared to the control. Furthermore, the increase in activity was higher in the case of desiccation stress and desiccation stress combined with heat stress than that in other singular and combinatorial stress exposure simultaneously (Figures 10A,B).

## 4 Discussion

**FIGURE 11:**
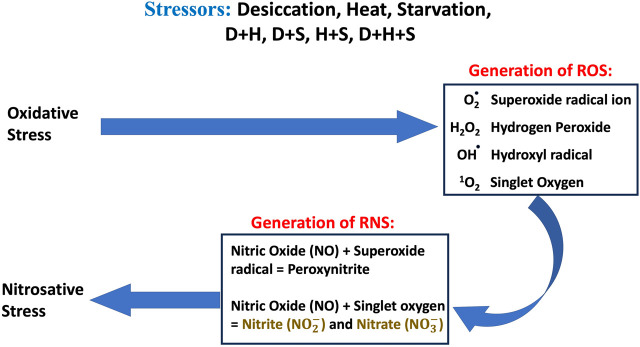
Schematic diagram of the overview of oxidative and nitrosative stress and generation of ROS and RNS.

The co-occurrence of heat, desiccation, and starvation as stressors has been well documented, and both plants and animals are equally vulnerable to such multi-stress environments. It is also well documented that in multi-stress environments, oxidative stress is one of the most common denominators of environmental stressors (Samet and Wages, 2018), and the occurrence of multiple stresses triggers an overproduction of ROS. Over a period, nitric oxide (NO) reacts rapidly with superoxide (O_2_^•−^), producing peroxynitrite (ONOO^−^) under excessive oxidation conditions, resulting in nitrosative stress. Under the nitrosative stress regime, additional RNS are generated (Figure 11). When the larvae of *D. melanogaster* were exposed to stressors either in singular or combinatorial form, the increase in RONS was evident, thereby confirming the convergence of desiccation stress, heat stress, and starvation stress in oxidative and nitrosative stress. The generation of ROS/RNS was always higher in the case of combinatorial stress than in singular exposure. The increased MDA concentration, protein carbonyl content, and AOPPs, damage markers of oxidative and nitrosative stress, further confirmed the biochemical and molecular signatures of the stress regime after desiccation stress, heat stress, and starvation stress, whether exposed individually or in combination. Previous reports of multi-stress environments have revealed that combinatorial responses are unique and cannot be extrapolated directly from the individual response to each type of stressor to which the organism is exposed. This might be due to the differential exposure of genes associated with the ROS gene network, as reported in *Arabidopsis* exposed to different stress treatments (Mittler et al., 2004; Mittler, 2006). In our study, stress-induced homeostasis could be ascertained through an increase in antioxidant activities. The antioxidant enzyme activity (SOD, CAT, and GPx) was higher, especially in combinatorial stress exposures, thereby suggesting how this defense system could manage oxidative and nitrosative stress, as well as cellular homeostasis. Other data on oxidative damage markers (MDA, protein carbonyl content, and AOPP) presented in this paper are analyzed, and desiccation was the most potent stressor, either acting individually or in combination with other stressors. Similar reports are available on other animals, where a combination of desiccation and heat stress can lead to greater lethal effects than those from different abiotic stress exposures (Jiang and Huang, 2001; Wang and Huang, 2004). Desiccation, as well as nutritional deprivation (starvation), can influence the thermal threshold of tolerance in insects by impacting homeostatic physiological mechanisms (Mitchell et al., 2017). In predatory mites, *Neoseiulus barkeri*, subjected to heat and desiccation stress, the mortality was mainly due to desiccation (Huang et al., 2019). Plants are also more vulnerable to desiccation-related stress while mitigating in a multi-stress environment. In *Brassica napus*, drought combined with heat stress led to more deleterious effects than drought alone. Moreover, in the presence of all three stressors, namely, drought + heat + nutrient deficiency, the damage is more than what might be due to a single stressor (Dikšaityte et al., 2020). In a seminal study on *D. melanogaster*, Bubliy et al. (2012) suggested that in a multi-stress environment, shared protective systems associated with plastic responses might be constrained. This study revealed that heat and desiccation hardening, along with acclimation to starvation, would incur metabolic costs, leading to reduced longevity (Bubliy et al., 2012). Our data on the total antioxidant enzyme activities (TAC) reflected a similar scenario. At the molecular level, several plants and animals respond through the expression of stress-responsive proteins like HSPs (Nguyen et al., 2017; Yurina, 2023). Ongoing proteomic studies in our laboratory on *Drosophila* under a multi-stress environment indicated the upregulation of HSPs (Bomble and Nath, 2019). The superoxide anion radical O_2_^•−^ is a major precursor of RONS production in cells, and an increase in this radical in a multi-stress environment ultimately leads to nitrosative stress (Ananda-Rivera et al., 2022). A higher level of O_2_^•−^ generates additional RNS like nitrite (NO_2_^-^) and nitrate (NO_3_^-^) in a continuum (Figure 11). A higher level of O_2_^•−^ and ONOO^−^ generates additional RNS like nitrite (NO_2_^-^) and nitrite (NO_3_^-^) in a continuum (Figure 11). Since energy availability plays a crucial role in an organism’s survival under a multi-stress environment, we focused on key mitochondrial enzymes involved in energy metabolism during the stress regime. We found the aconitase enzyme to be the best candidate for our present study because the aconitase enzyme is attacked by ROS and RNS. Aconitase activity in mitochondria has been reported to be a sensitive redox sensor of RONS (Tórtora et al., 2007; Lushchak et al., 2014). In the Krebs cycle, aconitase catalyzes citrate to isocitrate. However, during oxidative and nitrosative stress, aconitase is oxidized by nitric oxide (•NO), superoxide (O_2_^•−^), and peroxynitrite (ONOO^−^), generating inactive aconitase. In the present study, a significant inactivation of aconitase activity (Figure 9) was concomitant with the increase in the RONS level (Figure 8), both under singular and multiple stress regimes. Therefore, an increase in superoxide radical concentration, along with other RONS, might have led to the inactivation of the aconitase enzyme, as previously described (Liang et al., 1997; Miwa and Brand, 2005). Interestingly, the impact of desiccation stress was significantly higher than that of any other stressors impacting aconitase inactivation, whether exerting its effect singularly or in combination. This is the first report of multi-stressor-induced inactivation of aconitase through RONS in *Drosophila* or, for that matter, in any other terrestrial invertebrate animals. In *Drosophila*, the salivary gland provides an excellent experimental system for cellular, molecular, and physiological studies. The cells of salivary glands contain polytene nuclei and are metabolically active across all the developmental stages (Andrew et al., 2000). In the present study, we extended our biochemical experiments of ROS/RNS detection and quantification at the cellular level using salivary gland cells of *D. melanogaster* as the model tissue. Fluorescence microscopic detection of ROS (Figure 3) and RNS (Figure 6) in salivary gland cells corroborated biochemical findings, suggesting that a quantum of the multi-stress response was maximum during desiccation stress, as well as with other stressors in combination with desiccation. In recent literature, the impact of multiple stressors has been categorized into additive, synergistic, and antagonistic (Piggott et al., 2015; Simmons et al., 2021). If we compare the effects of two stressors, A and B, with the control C, the effect of A will be the change in the response above the baseline, represented as A−C. Similarly, the effect of B will be B−C. The stressor interaction will be “*additive*” if the sum of effects of A and B is above the baseline [response = (A + B) −C]. However, the interaction will be “*synergistic*” if the sum of the effects of A and B is greater than the sum of the effects of both treatments [response > (A + B)−C]. The impact of the simultaneous action of multiple stressors can be assessed using this paradigm (Fong et al., 2018) and conceptualized for inferring endpoints of multi-stress regimes (Piggott et al., 2015; Simmons et al., 2021). The findings of the present study revealed that the combinatory effect of stressors (D + H, H + S, D + S) was “*synergistic*” because the combined effect of two stressors was always greater than the sum of the effect when administered separately and was never equal (*additive*) to the mathematical summation of their impact when treated individually. This was evident in all the experimental results (Figures 1–6, 8, 10), except where the downregulation or inhibitory effect (*antagonistic*) served as a “proof of concept.”

Considering all the endpoints, the present study strongly suggested that multiple stress responses depended on the type of individual stressor and the combination of stressors, leading to a synergistic effect. Although molecular signaling pathways of abiotic stresses like desiccation, heat, and starvation converge at some point to the oxidative stress pathway, signaling pathways are different in abiotic stressors (Knight and Knight, 2001; Lacal, 2017). Future studies will reveal the intricacies of signaling networks of desiccation stress, heat stress, and starvation stress and key signaling molecules involved in cross-talk.

## 5 Conclusion

The study found that all three stressors caused oxidative and nitrosative stress responses. Desiccation stress was the most significant stressor compared to heat stress and starvation stress. When stressors were combined, D + H had a greater impact than other pairs of stressors. The findings suggest that the level of oxidative and nitrosative stress depends on the specific stressors and their combinations. The study highlights the importance of considering RNS as a stress marker, not just focusing on ROS generation. Research on RONS will provide a more comprehensive understanding of oxidative stress biology. The findings of this paper could lead to new research directions on managing and restoring the environment, using *Drosophila* as an indicator.

## Data Availability

The original contributions presented in the study are included in the article/Supplementary Material; further inquiries can be directed to the corresponding author.
