# Supplementary material for: Impact of singular versus combinatorial environmental stress on RONS generation in Drosophila melanogaster larvae
Source: Front Physiol. 2024 Aug 29;15:1426169. doi: 10.3389/fphys.2024.1426169 (PMC11420561; doi:10.3389/fphys.2024.1426169)
Supplement: Supplementary file 1 [file DataSheet1.PDF]

Supplementary data:

Stress Treatment	LT_{20} (Hrs)	Fiducial limits of LT_{20} value	LT_{50} (Hrs)	Fiducial limits of LT_{50} value	LT_{90} (Hrs)	Fiducial limits of LT_{90} value	Slope ± SE[b]	χ^2	d.f.[a]
Desiccation	8.092	7.520 - 8.707	9.494	8.823 - 10.016	11.391	10.889 - 11.767	12.131 ± 0.016	0.904	2
Heat	10.483	9.819 - 11.193	12.927	12.107 - 13.801	17.784	16.657 - 18.987	9.334 ± 0.015	0.994	9
Starvation	25.520	24.561- 26.517	30.263	29.098 - 31.416	39.139	37.668 - 40.668	11.505 ± 0.008	1.000	20
Desiccation +Starvation	8.092	7.520 - 8.707	9.494	8.823 - 10.016	11.391	10.889 - 11.767	12.131 ± 0.016	0.904	2
Desiccation + Heat	3.428	3.045 - 3.860	4.387	3.897 - 4.940	6.388	5.673 - 7.193	7.931 ± 0.026	0.775	3
Heat + Starvation	8.502	8.064 – 9.385	11.004	10.198 – 11.873	15.733	14.580 – 16.977	8.304 ± 0.017	1.000	8
Multiple Stress (D+H+S)	3.428	3.045 - 3.860	4.387	3.897 - 4.940	6.388	5.673 - 7.193	7.931 ± 0.026	0.775	3

Table 1: The LT_{20}, LT_{50}, LT_{90} values after stress treatment of *Drosophila melanogaster*, [a]Degrees of freedom; [b]Standard error; $\chi 2$=Chi-square value; LT_{20}=Lethal time required to kill 20% of test insect Larvae; LT_{50}=Lethal time required to kill 50% of test insect Larvae; LT_{90}=Lethal time required to kill 90% of test insect larvae.

Statistical analysis:

1. AOPP assay:

1way ANOVA Multiple comparisons						
1	Number of families	1				
2	Number of comparisons per family	6				
3	Alpha	0.05				
4						
5	Tukey's multiple comparisons test	Mean Diff.	95.00% CI of diff.	Significant?	Summary	Adjusted P Value
6						
7	C vs. D	-68.47	-86.74 to -50.21	Yes	***	0.0001
8	C vs. H	-20.14	-38.41 to -1.875	Yes	*	0.0347
9	C vs. S	1.497	-16.77 to 19.76	No	ns	0.9893
10	D vs. H	48.33	28.32 to 68.34	Yes	**	0.0011
11	D vs. S	69.97	49.96 to 89.98	Yes	***	0.0002
12	H vs. S	21.64	1.628 to 41.65	Yes	*	0.0374
13						

1way ANOVA Multiple comparisons						
1	Number of families	1				
2	Number of comparisons per family	10				
3	Alpha	0.05				
4						
5	Tukey's multiple comparisons test	Mean Diff.	95.00% CI of diff.	Significant?	Summary	Adjusted P Value
6						
7	C vs. D+H	-76.78	-92.35 to -61.21	Yes	****	<0.0001
8	C vs. H+S	-26.07	-41.64 to -10.5	Yes	**	0.0044
9	C vs. D+S	-58.52	-74.09 to -42.95	Yes	****	<0.0001
10	C vs. MS	-79.28	-94.85 to -63.71	Yes	****	<0.0001
11	D+H vs. H+S	50.72	33.66 to 67.77	Yes	***	0.0002
12	D+H vs. D+S	18.27	1.21 to 35.32	Yes	*	0.0374
13	D+H vs. MS	-2.5	-19.55 to 14.55	No	ns	0.9779
14	H+S vs. D+S	-32.45	-49.5 to -15.4	Yes	**	0.0022
15	H+S vs. MS	-53.22	-70.27 to -36.16	Yes	***	0.0001
16	D+S vs. MS	-20.77	-37.82 to -3.71	Yes	*	0.0211
17						

2. TBARS Assay:

	**1way ANOVA** Multiple comparisons					
1	Number of families	1				
2	Number of comparisons per family	6				
3	Alpha	0.05				
4						
5	Tukey's multiple comparisons test	Mean Diff.	95.00% CI of diff.	Significant?	Summary	Adjusted P Value
6						
7	C vs. D	-0.685	-0.9284 to -0.4416	Yes	**	0.0012
8	C vs. H	-0.5	-0.7434 to -0.2566	Yes	**	0.0039
9	C vs. S	-0.305	-0.5484 to -0.0616	Yes	*	0.0235
10	D vs. H	0.185	-0.0584 to 0.4284	No	ns	0.1142
11	D vs. S	0.38	0.1366 to 0.6234	Yes	*	0.0108
12	H vs. S	0.195	-0.0484 to 0.4384	No	ns	0.0984
13						

	**1way ANOVA** Multiple comparisons					
1	Number of families	1				
2	Number of comparisons per family	10				
3	Alpha	0.05				
4						
5	Tukey's multiple comparisons test	Mean Diff.	95.00% CI of diff.	Significant?	Summary	Adjusted P Value
6						
7	C vs. D+H	-1.065	-1.238 to -0.892	Yes	****	<0.0001
8	C vs. H+S	-0.711	-0.8558 to -0.5662	Yes	****	<0.0001
9	C vs. D+S	-0.69	-0.863 to -0.517	Yes	****	<0.0001
10	C vs. MS	-1.15	-1.323 to -0.977	Yes	****	<0.0001
11	D+H vs. H+S	0.354	0.2092 to 0.4988	Yes	***	0.0002
12	D+H vs. D+S	0.375	0.202 to 0.548	Yes	***	0.0005
13	D+H vs. MS	-0.085	-0.258 to 0.08804	No	ns	0.4851
14	H+S vs. D+S	0.021	-0.1238 to 0.1658	No	ns	0.9849
15	H+S vs. MS	-0.439	-0.5838 to -0.2942	Yes	****	<0.0001
16	D+S vs. MS	-0.46	-0.633 to -0.287	Yes	***	0.0001
17						

3. Protein Carbonyl content:

1way ANOVA<br>Multiple comparisons						
1	Number of families	1				
2	Number of comparisons per family	6				
3	Alpha	0.05				
4						
5	Tukey's multiple comparisons test	Mean Diff.	95.00% CI of diff.	Significant?	Summary	Adjusted P Value
6						
7	C vs. D	-0.685	-0.9284 to -0.4416	Yes	**	0.0012
8	C vs. H	-0.5	-0.7434 to -0.2566	Yes	**	0.0039
9	C vs. S	-0.305	-0.5484 to -0.0616	Yes	*	0.0235
10	D vs. H	0.185	-0.0584 to 0.4284	No	ns	0.1142
11	D vs. S	0.38	0.1366 to 0.6234	Yes	*	0.0108
12	H vs. S	0.195	-0.0484 to 0.4384	No	ns	0.0984

1way ANOVA<br>Multiple comparisons						
1	Number of families	1				
2	Number of comparisons per family	10				
3	Alpha	0.05				
4						
5	Tukey's multiple comparisons test	Mean Diff.	95.00% CI of diff.	Significant?	Summary	Adjusted P Value
6						
7	C vs. D+H	-0.2083	-0.2429 to -0.1738	Yes	****	<0.0001
8	C vs. H+S	-0.1333	-0.1679 to -0.09878	Yes	****	<0.0001
9	C vs. D+S	-0.1767	-0.2076 to -0.1458	Yes	****	<0.0001
10	C vs. MS	-0.2133	-0.2479 to -0.1788	Yes	****	<0.0001
11	D+H vs. H+S	0.075	0.03715 to 0.1129	Yes	**	0.0012
12	D+H vs. D+S	0.03167	-0.002886 to 0.06622	No	ns	0.0727
13	D+H vs. MS	-0.005	-0.04285 to 0.03285	No	ns	0.9876
14	H+S vs. D+S	-0.04333	-0.07789 to -0.008781	Yes	*	0.0168
15	H+S vs. MS	-0.08	-0.1179 to -0.04215	Yes	***	0.0008
16	D+S vs. MS	-0.03667	-0.07122 to -0.002114	Yes	*	0.0382

4. ROS:

1way ANOVA Multiple comparisons					
1	Number of families	1				
2	Number of comparisons per family	10				
3	Alpha	0.05				
4						
5	Tukey's multiple comparisons test	Mean Diff.	95.00% CI of diff.	Significant?	Summary	Adjusted P Value
6						
7	C vs. D+H	-66	-72.89 to -59.11	Yes	****	<0.0001
8	C vs. H+S	-47.33	-54.22 to -40.44	Yes	****	<0.0001
9	C vs. D+S	-45	-51.89 to -38.11	Yes	****	<0.0001
10	C vs. MS	-65.33	-72.22 to -58.44	Yes	****	<0.0001
11	D+H vs. H+S	18.67	12.5 to 24.83	Yes	****	<0.0001
12	D+H vs. D+S	21	14.84 to 27.16	Yes	****	<0.0001
13	D+H vs. MS	0.6667	-5.495 to 6.829	No	ns	0.9956
14	H+S vs. D+S	2.333	-3.829 to 8.495	No	ns	0.7126
15	H+S vs. MS	-18	-24.16 to -11.84	Yes	****	<0.0001
16	D+S vs. MS	-20.33	-26.5 to -14.17	Yes	****	<0.0001
17						

5. H_2O_2

1way ANOVA Multiple comparisons						
1	Number of families	1				
2	Number of comparisons per family	6				
3	Alpha	0.05				
4						
5	Tukey's multiple comparisons test	Mean Diff.	95.00% CI of diff.	Significant?	Summary	Adjusted P Value
6						
7	C vs. D	-58	-64.13 to -51.87	Yes	****	<0.0001
8	C vs. H	-45.33	-51.47 to -39.2	Yes	****	<0.0001
9	C vs. S	-24.67	-30.8 to -18.53	Yes	****	<0.0001
10	D vs. H	12.67	6.535 to 18.8	Yes	***	0.0008
11	D vs. S	33.33	27.2 to 39.47	Yes	****	<0.0001
12	H vs. S	20.67	14.53 to 26.8	Yes	****	<0.0001
13						

6. RONS

	1way ANOVA Multiple comparisons					
1	Number of families	1				
2	Number of comparisons per family	6				
3	Alpha	0.05				
4						
5	Tukey's multiple comparisons test	Mean Diff.	95.00% CI of diff.	Significant?	Summary	Adjusted P Value
6						
7	C vs. D	-63.67	-72.63 to -54.7	Yes	****	<0.0001
8	C vs. H	-40	-48.96 to -31.04	Yes	****	<0.0001
9	C vs. S	-25.33	-34.3 to -16.37	Yes	****	<0.0001
10	D vs. H	23.67	14.7 to 32.63	Yes	***	0.0001
11	D vs. S	38.33	29.37 to 47.3	Yes	****	<0.0001
12	H vs. S	14.67	5.704 to 23.63	Yes	**	0.0035

	1way ANOVA Multiple comparisons					
1	Number of families	1				
2	Number of comparisons per family	10				
3	Alpha	0.05				
4						
5	Tukey's multiple comparisons test	Mean Diff.	95.00% CI of diff.	Significant?	Summary	Adjusted P Value
6						
7	C vs. D+H	-92.67	-107 to -78.31	Yes	****	<0.0001
8	C vs. H+S	-56	-70.35 to -41.65	Yes	****	<0.0001
9	C vs. D+S	-63.67	-78.02 to -49.31	Yes	****	<0.0001
10	C vs. MS	-92.67	-107 to -78.31	Yes	****	<0.0001
11	D+H vs. H+S	36.67	22.31 to 51.02	Yes	****	<0.0001
12	D+H vs. D+S	29	14.65 to 43.35	Yes	***	0.0004
13	D+H vs. MS	0	-14.35 to 14.35	No	ns	>0.9999
14	H+S vs. D+S	-7.667	-22.02 to 6.687	No	ns	0.4451
15	H+S vs. MS	-36.67	-51.02 to -22.31	Yes	****	<0.0001
16	D+S vs. MS	-29	-43.35 to -14.65	Yes	***	0.0004

7. RNS:

	1way ANOVA<br>Multiple comparisons					
1	Number of families	1				
2	Number of comparisons per family	10				
3	Alpha	0.05				
4						
5	Tukey's multiple comparisons test	Mean Diff.	95.00% CI of diff.	Significant?	Summary	Adjusted P Value
6						
7	C vs. D+H	-1.883	-2.064 to -1.702	Yes	****	<0.0001
8	C vs. H+S	-1.29	-1.471 to -1.109	Yes	****	<0.0001
9	C vs. D+S	-1.72	-1.918 to -1.522	Yes	****	<0.0001
10	C vs. MS	-1.915	-2.113 to -1.717	Yes	****	<0.0001
11	D+H vs. H+S	0.5933	0.4314 to 0.7553	Yes	****	<0.0001
12	D+H vs. D+S	0.1633	-0.01775 to 0.3444	No	ns	0.0776
13	D+H vs. MS	-0.03167	-0.2128 to 0.1494	No	ns	0.9660
14	H+S vs. D+S	-0.43	-0.6111 to -0.2489	Yes	***	0.0004
15	H+S vs. MS	-0.625	-0.8061 to -0.4439	Yes	****	<0.0001
16	D+S vs. MS	-0.195	-0.3934 to 0.003372	No	ns	0.0539

	1way ANOVA<br>Multiple comparisons					
1	Number of families	1				
2	Number of comparisons per family	6				
3	Alpha	0.05				
4						
5	Tukey's multiple comparisons test	Mean Diff.	95.00% CI of diff.	Significant?	Summary	Adjusted P Value
6						
7	C vs. D	-1.747	-2.177 to -1.317	Yes	****	<0.0001
8	C vs. H	-0.9967	-1.427 to -0.5667	Yes	***	0.0003
9	C vs. S	-0.52	-0.95 to -0.09003	Yes	*	0.0198
10	D vs. H	0.75	0.32 to 1.18	Yes	**	0.0023
11	D vs. S	1.227	0.7967 to 1.657	Yes	****	<0.0001
12	H vs. S	0.4767	0.04669 to 0.9066	Yes	*	0.0308

8. Antioxidant enzymes:

1way ANOVA<br>Multiple comparisons						
1	Number of families	1				
2	Number of comparisons per family	10				
3	Alpha	0.05				
4						
5	Tukey's multiple comparisons test	Mean Diff.	95.00% CI of diff.	Significant?	Summary	Adjusted P Value
6						
7	Control vs. D+H	-57.33	-67.55 to -47.11	Yes	****	<0.0001
8	Control vs. H+S	-20.33	-30.55 to -10.11	Yes	***	0.0005
9	Control vs. D+S	-32.33	-42.55 to -22.11	Yes	****	<0.0001
10	Control vs. MS	-57.33	-67.55 to -47.11	Yes	****	<0.0001
11	D+H vs. H+S	37	26.78 to 47.22	Yes	****	<0.0001
12	D+H vs. D+S	25	14.78 to 35.22	Yes	****	<0.0001
13	D+H vs. MS	0	-10.22 to 10.22	No	ns	>0.9999
14	H+S vs. D+S	-12	-22.22 to -1.779	Yes	*	0.0206
15	H+S vs. MS	-37	-47.22 to -26.78	Yes	****	<0.0001
16	D+S vs. MS	-25	-35.22 to -14.78	Yes	****	<0.0001

1way ANOVA<br>Multiple comparisons						
1	Number of families	1				
2	Number of comparisons per family	6				
3	Alpha	0.05				
4						
5	Tukey's multiple comparisons test	Mean Diff.	95.00% CI of diff.	Significant?	Summary	Adjusted P Value
6						
7	C vs. D	-0.3418	-0.4643 to -0.2193	Yes	***	0.0006
8	C vs. H	-0.2063	-0.3288 to -0.08383	Yes	**	0.0060
9	C vs. S	-0.05683	-0.1793 to 0.06567	No	ns	0.4064
10	D vs. H	0.1355	0.0013 to 0.2697	Yes	*	0.0483
11	D vs. S	0.285	0.1508 to 0.4192	Yes	**	0.0021
12	H vs. S	0.1495	0.0153 to 0.2837	Yes	*	0.0333

1way ANOVA Multiple comparisons						
1	Number of families	1				
2	Number of comparisons per family	10				
3	Alpha	0.05				
4						
5	Tukey's multiple comparisons test	Mean Diff.	95.00% CI of diff.	Significant?	Summary	Adjusted P Value
6						
7	C vs. D+H	-0.5898	-0.8501 to -0.3296	Yes	***	0.0009
8	C vs. H+S	-0.3238	-0.5841 to -0.06361	Yes	*	0.0191
9	C vs. D+S	-0.3418	-0.6021 to -0.08161	Yes	*	0.0148
10	C vs. MS	-0.6433	-0.9036 to -0.3831	Yes	***	0.0005
11	D+H vs. H+S	0.266	-0.01906 to 0.5511	No	ns	0.0661
12	D+H vs. D+S	0.248	-0.03706 to 0.5331	No	ns	0.0864
13	D+H vs. MS	-0.0535	-0.3386 to 0.2316	No	ns	0.9482
14	H+S vs. D+S	-0.018	-0.3031 to 0.2671	No	ns	0.9991
15	H+S vs. MS	-0.3195	-0.6046 to -0.03444	Yes	*	0.0307
16	D+S vs. MS	-0.3015	-0.5866 to -0.01644	Yes	*	0.0395

1way ANOVA Multiple comparisons						
1	Number of families	1				
2	Number of comparisons per family	10				
3	Alpha	0.05				
4						
5	Tukey's multiple comparisons test	Mean Diff.	95.00% CI of diff.	Significant?	Summary	Adjusted P Value
6						
7	Control vs. D+H	-555.5	-655 to -455.9	Yes	****	<0.0001
8	Control vs. H+S	-356.5	-456 to -256.9	Yes	****	<0.0001
9	Control vs. D+S	-420.2	-519.7 to -320.7	Yes	****	<0.0001
10	Control vs. MS	-579.4	-685.8 to -473	Yes	****	<0.0001
11	D+H vs. H+S	199	106.9 to 291.1	Yes	***	0.0001
12	D+H vs. D+S	135.3	43.12 to 227.4	Yes	**	0.0036
13	D+H vs. MS	-23.92	-123.4 to 75.6	No	ns	0.9385
14	H+S vs. D+S	-63.75	-155.9 to 28.38	No	ns	0.2468
15	H+S vs. MS	-222.9	-322.4 to -123.4	Yes	****	<0.0001
16	D+S vs. MS	-159.2	-258.7 to -59.65	Yes	**	0.0018
